# Mechanisms of hyponatremia and diabetes insipidus after acute spinal cord injury: a critical review

**DOI:** 10.1186/s41016-023-00347-y

**Published:** 2023-11-15

**Authors:** Lianhua Li, Yanhui Guo, Chen Chen, Zhonghe Wang, Zhi Liu

**Affiliations:** Department of Orthopedics, the Fourth Medical Center of PLA General Hospital, Beijing, China

**Keywords:** Mechanism, Hyponatremia, Diabetes insipidus, Spinal cord injury

## Abstract

The incidence of hyponatremia after spinal cord injury was reported to be between 25 and 80%. Hyponatremia can lead to a variety of clinical symptoms, from mild to severe and even life-threatening. Hyponatremia is often associated with diabetes insipidus, which refers to insufficient arginine vasopressin (AVP) secretion or defective renal response to AVP, with clinical manifestations of syndromes such as hypoosmolality, polydipsia, and polydipsia. Recent mechanistic studies on hyponatremia and diabetes insipidus after acute spinal cord injury have been performed in isolation, without integrating the above two symptoms into different pathological manifestations that occur in the same injury state and without considering the acute spinal cord injury patient’s condition as a whole. The therapeutic principles of CSWS and SIADH are in opposition to one another. It is not easy to identify the mechanism of hyponatremia in clinical practice, which makes selecting the treatment difficult. According to the existing theories, treatments for hyponatremia and diabetes insipidus together are contraindicated, whether the mechanism of hyponatremia is thought to be CSWS or SIADH. In this paper, we review the mechanism of these two pathological manifestations and suggest that our current understanding of the mechanisms of hyponatremia and diabetes insipidus after high acute cervical SCI is insufficient, and it is likely that there are other undetected pathogenetic mechanisms.

## Background

Spinal cord injury (SCI) often leads to impaired somatosensory, motor, and autonomic nervous system function below the level of the injury. Though many neurorestorative treatments have been applied to restore damaged neurological functions and/or structures for patients with SCI, a variety of complications can still occur, the most common of which is hyponatremia [1]. Hyponatremia is defined as a serum sodium concentration < 135 mmol/L, and it occurs in up to 15 to 20% of patients admitted to the emergency department and in more than 20% of critically ill patients [2]. The incidence of hyponatremia after SCI was reported to be between 25 and 80% [3–5]. Hyponatremia can lead to a variety of clinical symptoms, from mild to severe and even life-threatening [6]. Hyponatremia can aggravate secondary SCI and affect patient rehabilitation, and early detection and correction of hyponatremia is essential to improve patient outcomes [7].

Hyponatremia is often associated with diabetes insipidus, which refers to insufficient arginine vasopressin (AVP) secretion or defective renal response to AVP, with clinical manifestations of syndromes such hypoosmolality, polydipsia, and polydipsia [8]. AVP is a neurohypophysial hormone synthesized within hypothalamus and transported to multiple areas, acting as a neurotransmitter/neuromodulator. Lin reported 17 patients with hyponatremia after SCI and 10 patients (58.82%) with diabetes insipidus [9]. Sun et al. reported 15 patients with complete SCI from C3 to C5 who developed hyponatremia and diabetes insipidus within day 5 to 12 after injury. After treatment, 13 patients showed improvement and two patients died [10]. If early treatment is improper in patients with hyponatremia complicated with diabetes insipidus after acute SCI, the patient’s condition can be aggravated, which may endanger their life and negatively affect their rehabilitation [11, 12].

Studies have found that the degree of blood sodium reduction in patients with acute cervical SCI and hyponatremia is related to the segment and degree of the SCI. The more severe the level of SCI, the lower the blood sodium concentration, and the more likely the patient is to have a worse level of polyuria [13, 14]. Recent mechanistic studies on hyponatremia and diabetes insipidus after acute SCI have been performed in isolation. In this paper, we reviewed the mechanism of these two pathological manifestations and tried to find out the intrinsic correlation of the pathological mechanisms for these two conditions.

## Mechanism of hyponatremia after acute SCI

**Fig. 1:**
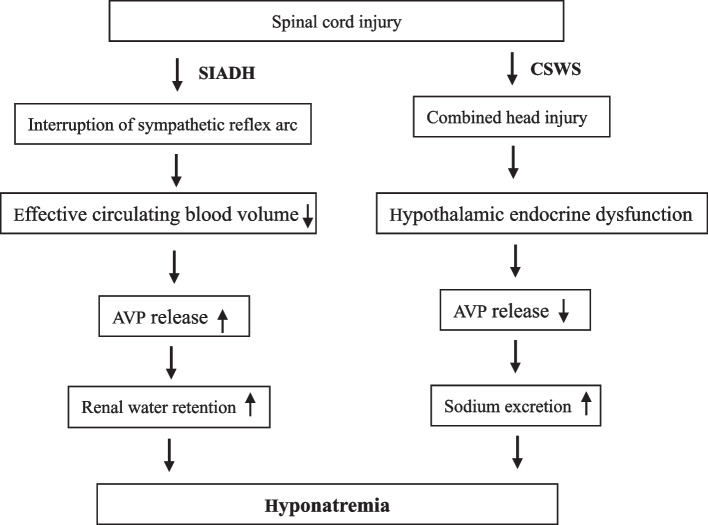
A flowchart to illustrate hyponatremia mechanisms

The mechanism of hyponatremia after SCI is complex and unclear. In addition to poor diet, application of diuretics and dehydrating agents, and combined craniocerebral trauma in early patients, hyponatremia after SCI is generally considered to be related to central nervous system dysfunction, including the syndrome of inappropriate antidiuretic hormone secretion (SIADH) and cerebral salt wasting syndrome (CSWS) [15, 16]. Both SIADH and CSWS are clinically characterized by hyponatremia, low plasma osmolality, and the accompanying neurological symptoms, but their blood volume, sodium metabolism, and treatment are different. SIADH refers to hypervolemic hyponatremia that is caused by abnormally increased AVP secretion, which promotes renal water retention and sodium excretion and results in increased urinary sodium and water retention. CSWS refers to hypovolemic hyponatremia, which is caused by excessive renal sodium excretion that results from hypothalamic endocrine dysfunction (Fig. 1) [5].

## The origin of the two conflicting theories

These two theories for hyponatremia, SIADH and CSWS, are contradictory as follows: SIADH is hypervolemic hyponatremia that is caused by water retention, while CSWS is hypovolemic hyponatremia that is caused by excessive sodium excretion; SIADH is the hypersecretion of antidiuretic hormone which also known as arginine vasopressin (AVP), while CSWS is the hyposecretion of AVP; and SIADH requires water restriction and CSWS requires fluid replacement therapeutically. There are two opposite mechanisms to explain the same pathological manifestation after acute SCI.

With the invention of the flame photometer, it became possible to determine serum sodium concentration. In 1950, Peters found that in patients with central nervous system diseases, urinary sodium loss remained in the presence of hyponatremia and a low-salt diet. They proposed the theory of CSWS, which is characterized by polyuria, increased urinary sodium, and dehydration [17]. In 1957, Schwartz et al. found that patients with lung cancer developed unexplained hyponatremia and their clinical features were similar to those of patients who had been taking pituitrin extract, which was considered to be the antidiuretic effect that was produced by AVP in pituitrin extract. This was the origin of the SIADH theory, which suggests that hyponatremia is caused by impaired renal drainage that results from the action of AVP [18]. In 1981, Nelson et al. investigated 12 patients who met the SIADH criteria and found that these patients also had significantly reduced blood volume, which was attributed to the mechanism of hyponatremia that was characterized by this hypovolemia that resulted from CSWS. They attributed the cause of hyponatremia to CSWS, characterized by elevated natriuretic peptide levels, a negative sodium balance, and decreased central venous pressure [19].

In summary, SIADH and CSWS were historically artificially divided on the basis of their theoretical hyponatremia mechanisms. Although these conditions are different theoretically, they are not easily distinguished from each other in clinical practice, especially in patients with high-level SCI, the sympathetic nervous system is completely interrupted. Because these patients have hypotension and bradycardia, which makes it more difficult to differentiate SIADH from CSWS.

## Difficulties in clinically differentiating between SIADH and CSWS

After 2016, Misra et al. [20] and Cui et al. [21] successively proposed the diagnostic criteria for CSWS and SIADH. The diagnostic criteria for CSWS are as follows: clinical manifestations of hypovolemia, such as hypotension, dry mucosa, tachycardia, or orthostatic hypotension; laboratory evidence of dehydration, such as elevated hematocrit, hemoglobin, serum albumin, or blood urea nitrogen; negative fluid balance; and central venous pressure (CVP, which describes the pressure of pressure of the right atrium and upper and lower vena cava thoracic segments) < 6 cmH_2_O. The diagnostic criteria for SIADH are as follows: no signs of hypovolemia; no laboratory evidence of dehydration; normal or positive fluid balance without weight loss; and CVP > 6 cmH_2_O [20, 21].

Changes in blood volume are a key factor in distinguishing CSWS from SIADH. Among the methods for assessing blood volume, the radioisotope dilution technique is the gold standard and commonly used methods include 51-chromium-labeled red blood cells and iodine-131 or iodine-125 labeled human serum albumin. These methods are currently only used for laboratory studies because of their high cost and the complex methods required for their use [22]. The method of clinically assessing blood volume is influenced by many factors, and subjective judgment can be erroneous when the difference is not obvious.

Plasma AVP and natriuretic peptide concentrations were once used to identify SIADH and CSWS. Both SIADH and CSWS have been implicated in the abnormal impermeable release of AVP; thus, measuring the concentration of AVP and its surrogate markers in serum to make a differential diagnosis has a limited role [23, 24]. In SIADH, natriuretic peptide levels increase when there is too much water in the arterial circulation [25]. However, the natriuretic peptide test index has a short half-life and is greatly affected by other factors, such as head injury, pulmonary infection, and advanced age, which cannot be used to distinguish SIADH from CSWS [21, 26].

In cases where laboratory test results cannot easily distinguish between CSWS and SIADH, diagnostic treatment was once considered to be an alternative. Water restriction is recommended to treat hyponatremia caused by simple SIADH [2], but this may cause cerebral vasospasm and cerebral hypoperfusion [27]; therefore, diagnostic water restriction is not a safe and feasible method to identify the type of hyponatremia.

## Unclear mechanism of diabetes insipidus after SCI

**Fig. 2:**
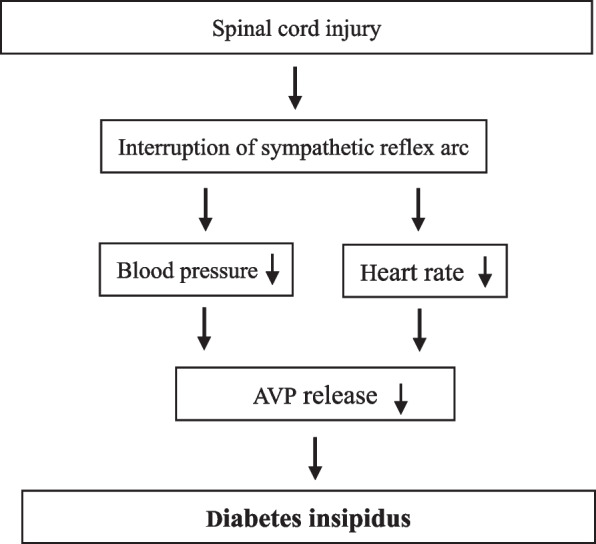
A flowchart to illustrate diabetes insipidus mechanism

Diabetes insipidus after SCI is common in patients with cervical and upper thoracic cord injury and rare in patients with lower thoracic and lumbar cord injury [28]. The current studies explain the mechanism of diabetes insipidus after SCI: sympathetic nerve interruption, loss of sympathetic innervation of the cardiovascular system, decreased blood pressure, and decreased heart rate. These factors cause decreased AVP secretion, resulting in polyuria (Fig. 2) [9–12]. This description is not compatible with a mechanism for regulating AVP production and secretion.

AVP secretion by the posterior pituitary is triggered by changes in plasma osmolality and effective circulating blood volume, which activate osmoreceptors [29] and baroreceptors [30], respectively. When serum sodium levels rise above 145 mmol/L, osmoreceptors in the hypothalamus are activated, which signals the supraoptic and paraventricular nuclei to secrete AVP into the blood via the posterior pituitary [31, 32]. Changes in thalamic magnocellular osmolality activate nonselective cation channels, increase the action potential firing rate, and trigger AVP release from axonal terminals [33, 34]. Elevated plasma AVP causes the kidney to reabsorb water, which dilutes the hypertonic sodium ions in the organism to reduce osmolality [28, 33]. Conversely, the decrease in osmotic pressure in vivo inhibits in vivo AVP secretion and promotes water excretion [35]. Changes in blood volume when blood pressure decreases, particularly when there is a decrease in effective circulating blood volume, can activate baroreceptors such that tonic inhibition of vagal afferent pathways is eliminated, which increases the non-osmotic release of AVP [32, 36]. Therefore, the decreased blood pressure does not decrease AVP secretion, and there must be another mechanism.

Some authors attributed diabetes insipidus after acute SCI to posterior pituitary dysfunction with a combined traumatic brain injury, resulting in AVP deficiency and thus diabetes insipidus [37, 38]. However, there was a lack of clinical evidence to support this claim. So the real mechanism of diabetes insipidus after SCI remains unclear.

## Existing theories cannot guide the treatment of hyponatremia and diabetes insipidus after acute SCI

The therapeutic principles of CSWS and SIADH are in opposition to one another. It is not easy to identify the mechanism of hyponatremia in clinical practice, which makes selecting the treatment difficult. The principles of treating CSWS are volume expansion, sodium supplementation, and, in severe cases, hormone replacement therapy [2, 39, 40]. The principle of treating SIADH is to limit fluid replacement and, in severe cases, to administer vasopressin V2 receptor antagonists [1, 41, 42].

According to the existing theories, treatments for both hyponatremia and diabetes insipidus are contraindicated, whether the mechanism of hyponatremia is thought to be CSWS or SIADH. Treatment of diabetes insipidus involves limiting fluid intake and supplementing AVP or its analogs to improve symptoms such as polyuria and thirst [8]. If hyponatremia is considered to be CSWS, volume expansion to treat CSWS is the opposite of fluid restriction, which is required to treat diabetes insipidus. If hyponatremia is considered to be SIADH, an AVP receptor antagonist should be administered to treat SIADH, but AVP should be administered to treat diabetes insipidus, which are opposite treatments.

## Future research direction

The serum AVP concentration sharply increased 15 min after spinal cord injury in an animal model. The differences were significantly observed compared to the control group [43]. However, the high secretion phase did not maintain such a long time. At 24 h after SCI, the urine output in the animal model significantly increased, and the concentration of serum AVP measured at 2 weeks after SCI significantly decreased [44, 45]. Research on the impact of SCI model in rats found that extensive activation of microglia occurred in the brain after SCI. It induced neuronal loss to a greater extent in the cortex, thalamus, and hippocampus at 12 weeks after SCI, which demonstrated that SCI would cause immune damage in the brain [46]. The immune damage of hypothalamic tissue after SCI may be a future research direction.

## Conclusions

In summary, the results can be summarized as follows: (1) hyponatremia and diabetes insipidus often occur together after high acute SCI, and they are still regarded as two different pathological manifestations with different mechanisms and not as a single condition; (2) the mechanisms of hyponatremia are explained as SIADH and CSWS, respectively, on the basis of historical factors; (3) SIADH and CSWS mechanisms are contradictory; (4) SIADH and CSWS mechanisms are difficult to differentiate clinically; (5) the pathogenesis of diabetes insipidus remains unclear, and interpretation of some studies is not consistent with the physiological mechanism; and (6) existing theories cannot guide clinical treatment, and dividing hyponatremia into CSWS and SIADH is contradictory to the treatment of diabetes insipidus. Thus, these findings suggest that our current understanding of the mechanisms of hyponatremia and diabetes insipidus after high acute cervical SCI is insufficient, and it is likely that there are other undetected pathogenetic mechanisms.

## Data Availability

Not applicable.

## Abbreviations

SCI: Spinal cord injury
AVP: Arginine vasopressin
SIADH: Syndrome of inappropriate antidiuretic hormone secretion
CSWS: Cerebral salt wasting syndrome
